# *N-*glycosylation of α_1D_-adrenergic receptor N-terminal domain is required for correct trafficking, function, and biogenesis

**DOI:** 10.1038/s41598-020-64102-4

**Published:** 2020-04-29

**Authors:** Eric M. Janezic, Sophia My-Linh Lauer, Robert George Williams, Michael Chungyoun, Kyung-Soon Lee, Edelmar Navaluna, Ho-Tak Lau, Shao-En Ong, Chris Hague

**Affiliations:** Department of Pharmacology, School of Medicine, University of Washington, 1959 NE Pacific Street, Seattle, WA 98185 USA

**Keywords:** G protein-coupled receptors, Membrane proteins, Glycosylation, Membrane trafficking, Post-translational modifications

## Abstract

G protein-coupled receptor (GPCR) biogenesis, trafficking, and function are regulated by post-translational modifications, including *N*-glycosylation of asparagine residues. α_1D_-adrenergic receptors (α_1D_-ARs) – key regulators of central and autonomic nervous system function – contain two putative *N*-glycosylation sites within the large N-terminal domain at N65 and N82. However, determining the glycosylation state of this receptor has proven challenging. Towards understanding the role of these putative glycosylation sites, site-directed mutagenesis and lectin affinity purification identified N65 and N82 as *bona fide* acceptors for *N*-glycans. Surprisingly, we also report that simultaneously mutating N65 and N82 causes early termination of α_1D_-AR between transmembrane domain 2 and 3. Label-free dynamic mass redistribution and cell surface trafficking assays revealed that single and double glycosylation deficient mutants display limited function with impaired plasma membrane expression. Confocal microscopy imaging analysis and SNAP-tag sucrose density fractionation assays revealed the dual glycosylation mutant α_1D_-AR is widely distributed throughout the cytosol and nucleus. Based on these novel findings, we propose α_1D-_AR transmembrane domain 2 acts as an ER localization signal during active protein biogenesis, and that α_1D_-AR N-terminal glycosylation is required for complete translation of nascent, functional receptor.

## Introduction

G protein-coupled receptors (GPCRs) are essential membrane proteins that regulate the vast majority of physiological functions in the human body. As a result, GPCRs have been estimated to be targeted by approximately one third of all currently approved medications^1^. Adrenergic receptors (ARs) are a clinically relevant subfamily of GPCRs. Activated by the endogenous sympathetic neurotransmitters epinephrine and norepinephrine, adrenergic GPCRs consist of three major subtypes: α_1_, α_2_, and β. The α_1_ sub-family – containing α_1A_, α_1B_, and α_1D_ subtypes^2^ – are targets for medications that regulate blood pressure^3,4^, bladder^5,6^, prostate^7,8^, and central nervous system function^9–11^. Thus, understanding the molecular and cellular mechanisms regulating α_1_-AR function will help spur the development of new medications associated with aberrant α_1_-AR signaling, such as hypertension, PTSD, schizophrenia, and benign prostatic hypertrophy^12–15^.

Among the three α_1_ subtypes, the α_1D_-AR remains poorly understood due to technical challenges. Relative to the closely related α_1A_ and α_1B_-AR subtypes, α_1D_-AR displays limited functional responses and minimal plasma membrane expression when expressed in heterologous cell culture^16–18^. Although pharmacologically detectable in intact isolated aortae in organ-tissue bath assays^19^, α_1D_-AR functional expression rapidly disappears in primary vascular smooth muscle cell cultures within 24–48 hours^20^. Also, immortalized cell lines that endogenously express α_1D_-ARs have yet to be discovered. Combined, these experimental clues indicate the molecular and cellular mechanisms governing α_1D_-AR functional expression in cells are unique amongst the α_1_-AR subtypes, and thus may be targeted by novel drugs to exogenously regulate α_1D_-AR signaling in human disease.

Important clues towards solving why α_1D_-ARs are poorly expressed in cell culture include two structural features distinct to the α_1D_-AR: (A) a C-terminal PSD-95/Dlg/ZO-1 (PDZ) ligand that ensures the formation of a modular, homodimeric macromolecular complex via binding to PDZ-domain containing proteins Scribble and syntrophin^21–25^; and (B) an atypical extracellular N-terminal domain (NTD). The average NTD for class A GPCRs is 40 amino acids^26^, making the 95 amino acid α_1D_ NTD unusually long. We, and others, have previously demonstrated the α_1D_-AR NTD contains an endoplasmic reticulum (ER) retention signal^17,18,27^, and that the NTD undergoes an endogenous cleavage event that enhances α_1D_-AR plasma membrane trafficking and agonist-stimulated functional responses^28^. Unfortunately, the mechanisms by which the NTD regulates α_1D_-AR trafficking and function are unknown.

GPCR trafficking is a highly complex process that is regulated in part by multiple factors, including Rab GTPases^29,30^, TBC domain-containing proteins^31^, GPCR oligomerization^32,33^, N-terminal cleavage^34–37^, and N-terminal translocation and glycosylation in the ER lumen^38–41^. Previous studies employed WGA lectin and deglycosylating enzymes to demonstrate endogenous α_1_-ARs are glycosylated in rat brain^42^, but were unable to determine if individual α_1_-AR subtypes were glycosylated due to technical limitations^43^. Subsequent studies have produced conflicting results^27,44–46^, and as a result, it remains unclear how NTD glycosylation regulates α_1_-ARs physiological function. Interestingly, the α_1D_-AR NTD contains two putative *N*-glycosylation sites located at N65 and N82^47^. In this study, we leverage SNAP-epitope tag labeling and label-free dynamic mass redistribution technology to show, for the first time, that the α_1D_-AR NTD is dual glycosylated, thereby ensuring proper biosynthesis and trafficking of nascent receptors.

## Results and Discussion

### N-terminal glycosylation is required for complete α_1D_-AR biogenesis

**Figure 1:**
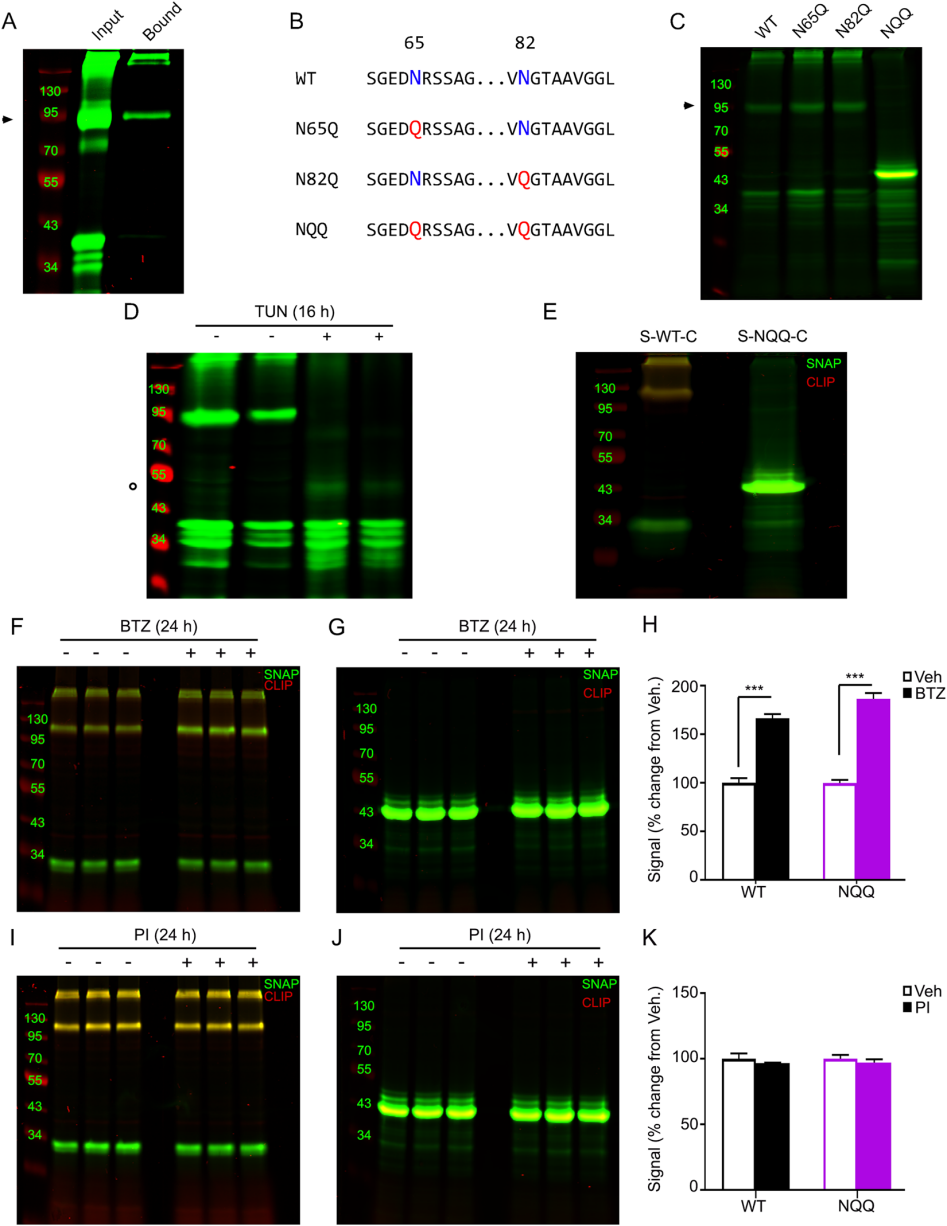
Site-directed mutagenesis and lectin affinity purification analyses reveal α_1D_-AR is dually glycosylated at N65 and N82. (**A**) Lysate from HEK293 cells expressing WT SNAP-α_1D_ (*input*) was incubated with lentil lectin sepharose beads to isolate glycosylated proteins. Bound protein was eluted with methyl-α-D-mannopyranoside (*bound*), and analyzed using PAGE NIR. SNAP-α_1D_ monomers (►) and higher order oligomers, as well as the previously described N-terminal cleavage product, are present in the elutant. (**B**) Schematic and (**C**) PAGE NIR of HEK293 cell lysate expressing WT, single glycosylation mutants (N65Q and N82Q), and double glycosylation mutant (NQQ) SNAP-α_1D_ species. (**D**) HEK293 cells expressing WT SNAP-α_1D_ were incubated for 16 hr with vehicle (−) or tunicamycin (TUN, +), and analyzed with PAGE NIR. A signal at 43 kDa was observed in the tunicamycin treated samples (○). (**E**) PAGE NIR of HEK293 cell lysate transfected with N-terminal SNAP-epitope (*λ* = 800 *nm, green*) and C-terminal CLIP-epitope (*λ* = 700 *nm, red*) dual tagged WT (S-WT-C) and NQQ (S-NQQ-C) α_1D_-AR constructs. (**F,I**) PAGE NIR of HEK293 cell lysate expressing S-WT-C or (**G,J**) S-NQQ-C following 24 hr bortezomib (BTZ) (**F,G**) or protease inhibitor (PI) treatment (**I,J**). (**H**) Quantitation of signals from F and G normalized to vehicle. (**K**) Quantitation of fluorescent signals from I and J normalized to vehicle. All gels are representative images from n = 3 experiments. For F and G, data are represented as mean ± SEM; Unpaired t tests, ***p < 0.001.

The α_1D_-AR N-terminal contains two putative *N*-glycosylation sites (N65, N82) with both serving as theoretical acceptors for *N*-glycans within the ER lumen^47^. *N*-glycosylation is the covalent attachment of an *N-*glycan sugar moiety to an asparagine residue within the consensus sequence N-X-S/T, where X is any amino acid except proline^48,49^. Thus, we sought to examine the glycosylation state of full length α_1D_-AR using PNGase F deglycosylation assays. To test this possibility, HEK293 cells were transiently transfected with N-terminal SNAP-epitope tagged α_1D_-AR cDNA constructs (SNAP-α_1D_). We have previously demonstrated the SNAP epitope-tag facilitates visual analysis of GPCR protein bands directly within polyacrylamide gels, and do not require nitrocellulose paper transfer or antibody staining, thus removing all potential false positive bands^22,28,50^. Incorporating this powerful technology, HEK293 cell lysates expressing SNAP-α_1D_ were lysed, denatured, and incubated with PNGase F, then subjected to polyacrylamide gel electrophoresis and near-infrared imaging (PAGE NIR). Similar to previous reports^27,46^, our results were inconclusive, likely due to instability of α_1D_-AR in the required buffer conditions (Supplementary Fig. S1A). To overcome this technical issue, we utilized lentil lectin affinity purification. Lentil lectin recognizes complex glycans containing α-(1 → 6)-linked fucose on the core GalNAc as well as glucose and/or α-mannose residues, and is active in a variety of buffer conditions^51,52^. HEK293 cells were transiently transfected with SNAP-α_1D_ and lysates were incubated with lentil lectin sepharose beads. Samples were eluted and subjected to PAGE NIR analysis. Shown in Fig. 1A are the results. In agreement with our previous studies^22,28^, the input lane demonstrates full length SNAP-α_1D_ is robustly expressed as a monomeric band at ~80 kDa, a larger, more intense band at ~90 kDa (Fig. 1A, *arrow*, Supplementary Fig. S1B), as higher order oligomers (MW > 180 kDa), and as multiple NTD cleavage products (MW = ~30–35 kDa). Remarkably, both ~90 kDa monomeric and multimeric SNAP-α_1D_ species were detected in the lectin bound lane (Fig. 1A, bound). Although faint, the largest α_1D_ NTD cleavage product^28^ was also observed in the lectin-bound sample. Thus, this experiment clearly demonstrates, for the first time, that the α_1D_ NTD is *N*-glycosylated.

Towards our goal of addressing the importance of each NTD glycosylation site for α_1D_-AR function, we created single (N65Q or N82Q) and double (NQQ) glycosylation deficient SNAP-α_1D_ mutants using PCR site-directed mutagenesis (see Fig. 1B for schematic). To ensure each α_1D_-AR NTD mutant was expressed as protein, cDNA constructs were transfected into HEK293 cells and subjected to PAGE NIR analysis. Both the N65Q and N82Q SNAP-α_1D_ NTD mutants display equivalent protein band patterns as SNAP-α_1D_ (Fig. 1C). Unexpectedly, the NQQ SNAP-α_1D_ double mutant did not produce monomeric or higher order oligomeric bands. Instead, NQQ SNAP-α_1D_ was primarily expressed as a single, robust band of ~43 kDa in size. Subtracting the size of the SNAP-epitope tag plus linker (25 kDa) yields a polypeptide of 18 kDa, ‒ roughly equivalent in size to the α_1D_ NTD, transmembrane domain (TM) 1, intracellular loop 1, and TM2. Subsequent lectin-purification assays reveal full length, N65Q and N82Q, but not NQQ, SNAP-α_1D_ species are glycosylated (Supplementary Fig. S1C). To ensure this unexpected NQQ product was due to inhibition of glycosylation, and not a by-product of mutation, cells expressing WT SNAP-α_1D_ were treated with tunicamycin – an inhibitor of *N*-glycosylation^53^. 24 hours after transfection with WT SNAP-α_1D_, HEK293 cells were treated with fresh media supplemented with 5 μg/mL tunicamycin or EtOH vehicle followed by PAGE NIR (Fig. 1D). Interestingly, though faint, the same ~43 kDa species observed in the NQQ SNAP-α_1D_ is also present in the tunicamycin treated samples (Fig. 1D; *circle*). Thus, this initial round of experiments demonstrates that (A) α_1D_-AR is glycosylated at N65 and N82; (B) only a single glycosylation site needs to be available for the NTD to become glycosylated and full length α_1D_ protein processing to occur; and (C) removal of both α_1D_ NTD glycosylation sites not only prevents glycosylation, but produces an abnormally short, previously unreported α_1D_-AR peptide species.

We next tested two potential explanations for this serendipitous, intriguing result: (A) the NQQ double mutation introduces a destabilizing effect, causing the α_1D_-AR to be targeted for degradation, with the observed 43 kDa band representing the major degradation product; or (B) NQQ is inhibiting proper translation of α_1D_-AR, causing an early termination after TM2. These hypotheses were tested using a dual epitope-tagging approach. InFusion PCR was used to add C-terminal CLIP-epitope tags to WT SNAP-α_1D_ (S-WT-C) and NQQ SNAP-α_1D_ (S-NQQ-C). CLIP is a homolog of SNAP that covalently interacts with benzylcytosine conjugates, displaying no cross-reactivity for the SNAP substrate, benzylguanine^54^. We reasoned that if A is true, CLIP substrate fluorescence in the 700 channel (red) would be observed in both the S-WT-C and S-NQQ-C PAGE NIR lanes. Conversely, we would expect to detect no 700 signal in the S-NQQ-C lane if B were true, as the CLIP tag would not be transcribed if α_1D_-AR translation was halted at TM2. Thus, S-WT-C and S-NQQ-C α_1D_-AR cDNA constructs were expressed in HEK293 cells and subjected to PAGE NIR analysis. Fig. 1E shows that overlapping CLIP (red) and SNAP (green) substrate signals are detectable in the S-WT-C lane (left). Contrarily, no CLIP signal is observed in the S-NQQ-C lane, and only the previously observed 43 kDa SNAP-α_1D_ species (Supplementary Fig. S1D).

As an orthogonal approach, HEK293 cells expressing either S-WT-C or S-NQQ-C were incubated with bortezomib (BTZ) ‒ a proteasomal inhibitor (Fig. 1F,G) ‒ or protease inhibitor (PI) cocktail (Fig. 1I,J) for 24 hours followed by PAGE NIR analysis. As expected, significant increases of S-WT-C and S-NQQ-C protein bands were observed with BTZ treatment (Fig. 1H; S-WT-C = 166.3 ± 4.3%, mean ± SEM of vehicle; S-NQQ-C = 186.3 ± 6.0% mean ± SEM of vehicle; Unpaired t test; p < 0.001), but not with PI cocktail treatment (Fig. 1K; S-WT-C = 96.7 ± 0.3%, mean ± SEM of vehicle; S-NQQ-C = 97.0 ± 2.5%, mean ± SEM of vehicle; Unpaired t test, p > 0.05). However, neither BTZ nor PI cocktail had any discernable effect on the molecular weight of the NQQ band; nor were CLIP signals observed in either condition. Taken together, these findings indicate that the NQQ α_1D_-AR species is not created by proteolytic cleavage and/or degradation of full-length α_1D_-AR.

**Figure 2:**
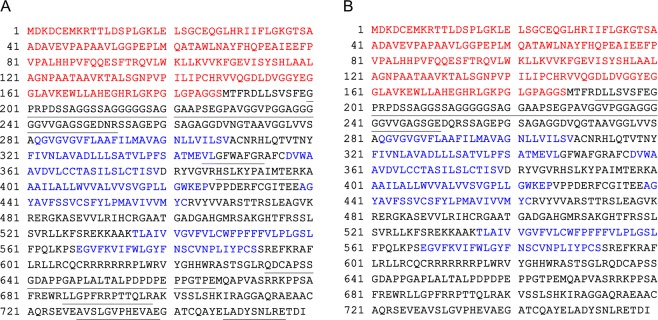
SNAP MS/MS analysis identifies distal peptides in WT, but not NQQ SNAP-α_1D_ lysates. (**A**) WT or (**B**) NQQ SNAP-α_1D_ was purified from HEK293 lysate using SNAP-Capture pull-down resin, then subjected to on-bead double-enzymatic digestion, and MS/MS analysis. Red text indicates SNAP-epitope tag. Blue text indicates transmembrane domains. Peptides identified in MS/MS analysis are underlined. See Supplementary Datas S1 and S2 for complete data set.

To further confirm the identity of this unexpected NQQ species, HEK293 cells were transiently transfected with either WT SNAP-α_1D_ or NQQ SNAP-α_1D_, lysed, and SNAP-fusion proteins were isolated with SNAP-Capture beads. Due to the covalent nature of the SNAP-Capture:SNAP-tag, an on-bead digest was performed using Trypsin and Glu-C proteases. Samples were subjected to MS/MS analysis (SNAP MS/MS). As shown in Fig. 2A, identified peptides spanned the entirety of the WT SNAP-α_1D_ (Fig. 2A, *underlined*). Contrarily, only peptides in the N-terminal domain were identified in NQQ SNAP-α_1D_ samples (Fig. 2B, *underlined*). Furthermore, previously reported α_1D_-AR interactors syntrophin^21,24,25^, members of the dystrophin-associated protein complex^23^, and scribble^22,25^ were identified in the WT, but not NQQ samples (Supplementary Datas S1, S2). Together, these data provide compelling evidence that glycosylation of both N65 and N82 are necessary for proper biogenesis of full-length α_1D_-AR, and disruption of these essential glycosylation sites results in early termination of α_1D_-AR processing after TM2.

### Glycosylation imparts α_1D_-AR function and plasma membrane insertion

**Figure 3:**
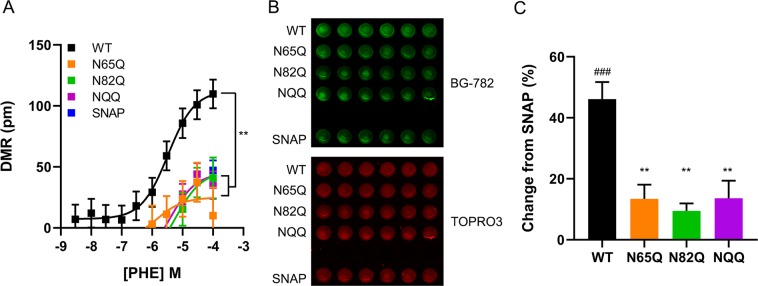
α_1D_-AR function and plasma membrane insertion is glycosylation dependent. (**A**) Dynamic mass redistribution assays quantifying phenylephrine efficacy in HEK293 cells transfected with WT, N65Q, N82Q, or NQQ SNAP-α_1D_. Data are the mean of 8 replicates ± SEM; **p < 0.01 E_max_ from WT. (**B**) Cell surface expression of WT, N65Q, N82Q, or NQQ SNAP-α_1D_ in fixed HEK293 cells labeled with the cell impermeable SNAP substrate, BG782 (*top panel, green*); nuclear stain, TO-PRO-3 was used to normalize for cell numbers (*bottom panel, red*). (**C**) Fluorescence intensity of data from B was normalized to cells expressing SNAP alone. Data are mean of 6 replicates ± SEM; One-way ANOVA with Tukey’s multiple comparisons post-hoc tests, **p < 0.01 from WT SNAP-α_1D_; ^###^p < 0.001 from empty SNAP.

The effects of NTD glycosylation on GPCR function and trafficking are highly divergent. Mutating N-terminal glycosylation sites decreases functional responses of the FSH^55^, dopamine D2^56^, and neurokinin 1 receptor subtypes^57^, while loss of glycosylation has no effect on the function of the histamine H2 receptor^58^. Conversely, blocking N-terminal glycosylation increases binding site density of the human oxytocin receptor^59^, and signaling efficacy of the vasopressin 1A receptor^60^. To understand how *N*-glycosylation impacts α_1D_-AR function, label-free dynamic mass redistribution (DMR) assays were used to quantify the efficacy of the α_1_-AR agonist phenylephrine for stimulating α_1D_ NTD glycosylation site mutants. HEK293 cells expressing WT, N65Q, N82Q, or NQQ SNAP-α_1D_ were seeded in 384-well DMR plates and incubated with increasing concentrations of phenylephrine to facilitate concentration-response curve analysis (Fig. 3A). Surprisingly, phenylephrine maximal responses for N65Q (24.99 ± 11.35 pm, mean ± SEM), N82Q (46.64 ± 9.96 pm, mean ± SEM), and NQQ (45.20 ± 8.35 pm, mean ± SEM) were significantly lower than WT SNAP-α_1D_ (112.5 ± 9.27, mean ± SEM; p < 0.01, One-way ANOVA with Tukey’s multiple comparisons post-hoc test).

Glycosylation has been shown to facilitate plasma membrane trafficking of the angiotensin II type 1^61^, GPR30^62^, rhodopsin 1^63^, δ-opioid receptor^40,64,65^, and P2Y_2_ receptor subtypes^66^. Therefore, one possible explanation for the reduced function of α_1D_ NTD glycosylation mutants may be aberrant cellular trafficking, leading to a decrease in cell surface expression. This was examined by quantifying WT, N65Q, N82Q and NQQ SNAP-α_1D_ plasma membrane expression levels in fixed HEK293 cells treated with the cell-impermeable SNAP substrate, BG-782 (Fig. 3B,C)^22,28,50^. Cells were also treated with nuclear stain TO-PRO-3 to normalize for cell number. We observed significant reductions in N65Q (13.40 ± 4.65%, mean ± SEM change from SNAP), N82Q (9.49 ± 5.95%, mean ± SEM change from SNAP), and NQQ (13.64 ± 5.76%, mean ± SEM change from SNAP) cell surface expression in comparison to WT SNAP-α_1D_ (46.13 ± 5.61%, mean ± SEM change from SNAP; p < 0.01, One-way ANOVA with Tukey’s multiple comparisons post-hoc test). Combined, these data strongly indicate both N65 and N82 must be glycosylated to facilitate α_1D_-AR plasma membrane insertion and agonist-stimulated functional responses in cultured human cells.

### TM2 of α_1D_-AR triggers ER translocation during protein synthesis

TM1 domain is thought to provide the ER localization signal for myriad polytropic integral membrane proteins ‒ including some GPCRs ‒ during protein synthesis^38^. Though, synthesis of TM2 has also been shown to trigger ribosomal translocation to the ER for some multi-pass transmembrane proteins, such as Cig30^67,68^ and ProW^69^. Because the α_1D_-AR NQQ mutant appears to cause early termination after TM2 (Figs. 1C,E,G,J and 2), we hypothesized that TM2 acts as the ER localization signal for α_1D_-ARs. To test this, we utilized two orthogonal, but complementary, approaches: sucrose density gradient and confocal imaging.

**Figure 4:**
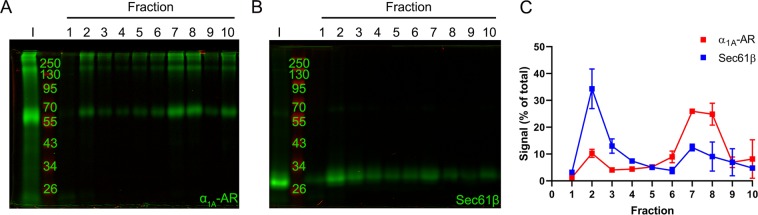
Sucrose density centrifugation of SNAP-epitope tagged plasma membrane and endoplasmic reticulum markers. HEK293 cell lysates expressing (**A**) SNAP-α_1A_-AR (*plasma membrane)* or (**B**) SNAP-Sec61β (*endoplasmic reticulum*) were labeled with SNAP substrate, BG-782, fractionated by discontinuous sucrose density gradient centrifugation, and analyzed using PAGE NIR. I = input. (**C**) Quantification of fluorescence signal from A and B normalized to respective inputs. Data are mean of 2 experiments ± SEM.

Previous studies examining α_1_-AR subcellular localization used cell fractionation/sucrose density gradient to sequester distinct cellular compartments, and then radioligand binding to quantify the number of receptors present in each compartment sample^18^. Although useful, this method is only able to detect properly folded, functional receptors that are able to bind ligand; and has non-optimal signal-to-noise ratios^18^. Thus, sucrose density centrifugation protocols were modified to incorporate the sensitivity of SNAP-epitope tag PAGE NIR imaging analysis. This novel experimental approach allows accurate detection of poorly expressing α_1D_-AR peptide species, regardless of their structural or functional state. Furthermore, the use of the SNAP epitope tag displays increased sensitivity compared to traditional immunoblotting techniques, which can be limited by the inability of antibodies to detect low expression levels of endogenous protein markers^70^. Thus, HEK293 cells were transfected with SNAP-α_1A_-AR, which we have previously shown expresses readily at the plasma membrane^18^, or SNAP-Sec61β, an ER integral membrane protein^71^. Cells were lysed in detergent free buffer then conjugated to SNAP substrate BG-782. Labelled lysates were then fractionated in a discontinuous gradient (see methods for details), collected, and subjected to PAGE NIR analysis. In each case, the detectable SNAP signal from each isolated fraction was normalized to input. Data were analyzed by area under curve (AUC) to quantify the distribution of each SNAP protein in specific fractions. Figure 4 displays the PAGE NIR band pattern for SNAP-α_1A_ (Fig. 4A, Supplementary Fig. S2A) and SNAP-Sec61β (Fig. 4B, Supplementary Fig. S2B). Subsequent AUC analysis revealed SNAP-Sec61β to be primarily distributed in fractions 1 through 4 with a peak in fraction 2 (91.50% total AUC; Fig. 4C), which is considered to be the ER fraction^18^. Conversely, SNAP-α_1A_ is significantly concentrated in fractions 6 through 9 with the maximum signal in fraction 7 (100% total AUC).

**Figure 5:**
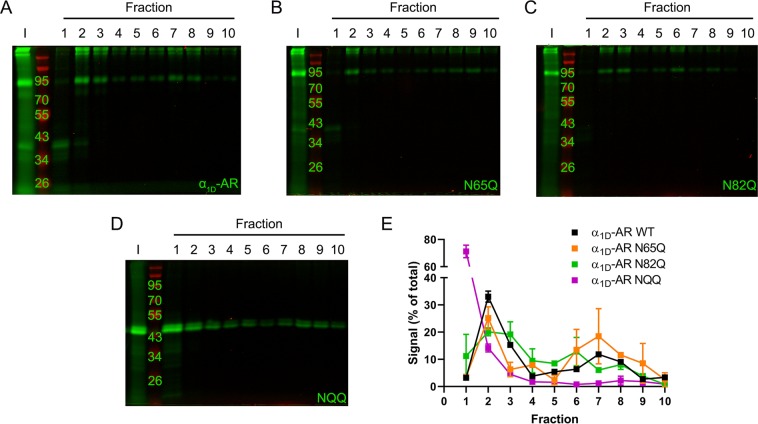
Sucrose density centrifugation of WT and glycosylation deficient SNAP-α_1D_ constructs. Representative PAGE NIR of HEK293 cell lysates transfected with (**A**) WT, (**B**) N65Q, (**C**) N82Q, or (**D**) NQQ SNAP-α_1D_ following sucrose density centrifugation. I = input. (**E**) Quantification of band intensity from A-D normalized to respective inputs. Data are presented as mean of 3 experiments ± SEM.

Next, WT, N65Q, N82Q and NQQ SNAP-α_1D_ cDNA constructs were examined (Fig. 5A–D). As expected based on the findings of previous studies performed by us and others^16–18^, WT SNAP-α_1D_ (Fig. 5A, Supplementary Fig. S3A) displayed a similar distribution pattern as SNAP-Sec61β, with a major peak spanning from fractions 1 to 4 (92.47% total AUC; Fig. 5E) and a minor peak in fractions 6 to 8 (7.53% total AUC). Similarly, N65Q SNAP-α_1D_ (Fig. 5B, Supplementary Fig. S3B) was bi-modally distributed, with peaks in fractions 1 to 3 (46.33% total AUC) and fractions 5 to 7 (53.67% total AUC; Fig. 5E). N82Q SNAP-α_1D_ (Fig. 5C, Supplementary Fig. S3C) was largely concentrated in fractions 1 through 4 with the maximum signal in fraction 2 (89.13% total AUC). A minor peak was also observed in fractions 6 to 7 (10.87% AUC; Fig. 5E). Remarkably, NQQ SNAP-α_1D_ (Fig. 5D, Supplementary Fig. S3D) formed a single, strong peak spanning fractions 1 to 3, with the majority of the protein concentrated to the first fraction (100% total AUC; Fig. 5E), which corresponds with a primarily cytosolic localization.

**Figure 6:**
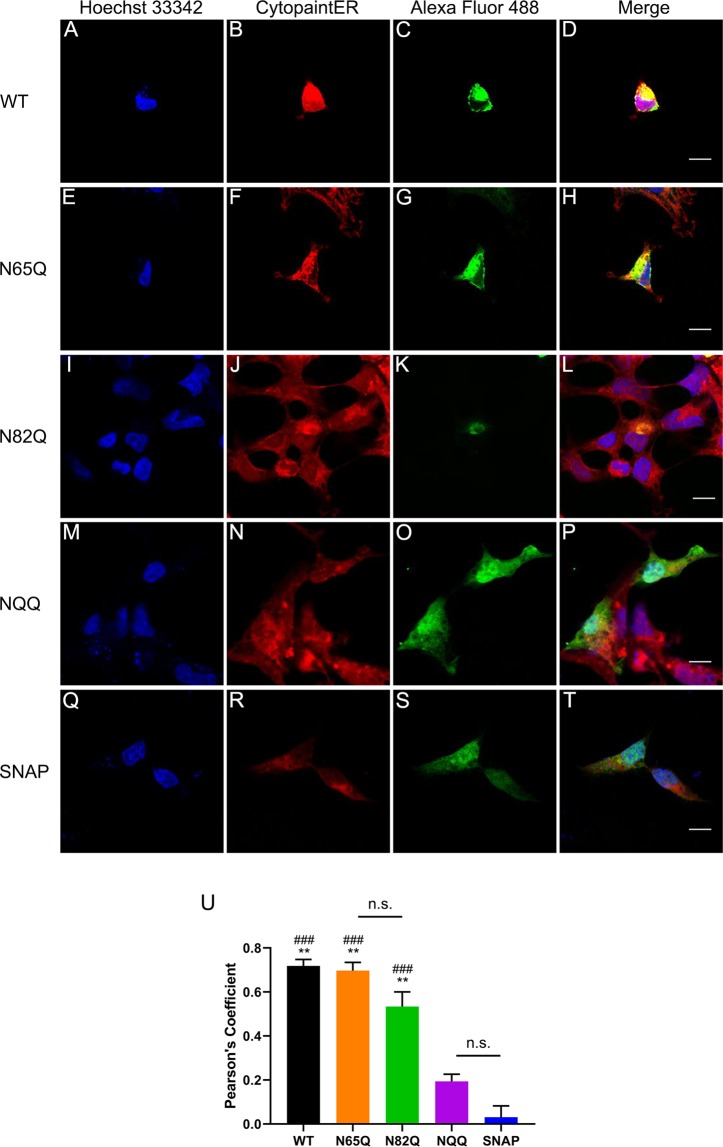
Confocal imaging reveals NQQ SNAP-α_1D_ is localized to cytosol and nucleus in HEK293 cells. HEK293 cells transfected with (**A–D**) WT, (**E–H**) N65Q, (**I–L**) N82Q, (**M–P**) NQQ SNAP-α_1D_, or (**Q–T**) empty pSNAP vector were fixed, stained for Hoechst 33342 (*blue*), fluorescence-Cytopainter stain (*red*), and SNAP-epitope tag (*BG-488; green*), and imaged using confocal microscopy. Right panels are merged images of three channels. Scale bar = 10 μm. (**U**) Pearson’s coefficients of SNAP and ER fluorescence signals were computed to measure the extent of colocalization. Data are mean of 5–6 cells ± SEM; One-way ANOVA with Tukey’s multiple comparisons post-hoc tests, ^###^p < 0.001 compared to empty SNAP; **p < 0.01 compared to NQQ SNAP-α_1D_, n.s = p > 0.05.

These findings were subsequently corroborated with confocal microscopy imaging analysis. HEK293 cells were transiently transfected with WT (Fig. 6A–D), N65Q (Fig. 6E–H), N82Q (Fig. 6I–L), NQQ (Fig. 6M–P) SNAP-α_1D_, or empty pSNAP vector (SNAP; Fig. 6Q–T). Cells were fixed with paraformaldehyde and detergent-permeabilized, then incubated with SNAP substrate Alexa Fluor 488 to label SNAP proteins and red fluorescence-Cytopainter stain to label the ER. Visual analysis reveals robust puncta of WT (Fig. 6D), N65Q (Fig. 6H), and N82Q (Fig. 6L) SNAP-α_1D_ within the ER. Contrarily, both NQQ SNAP-α_1D_ (Fig. 6P) and SNAP (Fig. 6T) are diffuse throughout the cell cytosol and nucleus. Colocalization quantification analysis (Fig. 6U) revealed a significant correlation between WT (Pearson’s coefficient = 0.72 ± 0.03, mean ± SEM, p < 0.001 from SNAP, p < 0.01 from NQQ), N65Q (Pearson’s coefficient = 0.70 ± 0.04, mean ± SEM, p < 0.001 from SNAP, p < 0.01 from NQQ), and N82Q (Pearson’s coefficient = 0.53 ± 0.07, mean ± SEM, p < 0.001 from SNAP, p < 0.01 from NQQ) SNAP-α_1D_ constructs with ER stain CytoPainter, when compared with NQQ SNAP-α_1D_ (Pearson’s coefficient = 0.19 ± 0.03, mean ± SEM, p = 0.12 from SNAP) and SNAP (Pearson’s coefficient = 0.03 ± 0.05, mean ± SEM). Together, our cell fractionation and confocal microscopy data support the hypothesis that α_1D_-AR requires the synthesis of TM2 prior to ER localization, where the N-terminal translocates into lumen, becomes glycosylated, and translation continues. If this does not occur, translation is prematurely terminated and the non-functional polypeptide is likely degraded via the ER-associated degradation (ERAD) pathway^71–74^. Further studies are necessary to determine the extent to which this mechanism is involved in the turnover of nascent α_1D_-AR and other GPCRs.

## Conclusion

Our findings support a model in which TM2, not TM1, triggers ribosomal translocation to the ER during α_1D_-AR synthesis^67–69,75^. Upon docking with the ER, the N-terminus is translocated into the ER lumen – possibly via the ER protein complex^38,76–78^ – where glycosylation occurs. This event prevents the N-terminus from retrotranslocating back to the cytosol, which anchors the nascent peptide in the ER membrane in the proper membrane topology, such that the N-terminal will be within the extracellular matrix upon plasma membrane insertion. This event is required before complete translation of the nascent polypeptide continues. However, if glycosylation is prevented, the immature receptor does not anchor in the ER membrane, thus terminating receptor translation after TM2 (see Supplementary Fig. S4 for schematic); and presumably this degenerate polypeptide is degraded via ERAD^73^ or other cytosolic degradation mechanisms^40,79^. Furthermore, we show that glycosylation of both N65 and N82 is required for proper function and plasma membrane expression of α_1D_-ARs.

## Materials and Methods

### Plasmids and chemicals

Molecular cloning was performed using inFusion HD cloning technology (Clontech/Takara Biotech, Mountain View, CA). The pSNAP_f_ and pCLIP vector, as well as SNAP substrates, BG-782 and Alexa Fluor 488, and CLIP substrate, BC-680 were purchased from New England Biolabs (Ipswich, MA). PageRuler Prestained NIR Protein Ladder was used for all PAGE NIR analyses (Thermo Fisher Scientific, Waltham, MA).

### Cell culture

Human Embryonic Kidney (HEK) 293 cells were grown in Dulbecco’s Modified Eagle’s Medium (Corning, Corning, NY) supplemented with 10% fetal bovine serum and 2 mM L-glutamine at 37 °C in 5% CO_2_. Cells were used ~48 hrs post-transfection with polyethyleneimine unless stated otherwise.

### PNGase assay

Cells were lysed in 20 mM Tris-HCl (pH 8), 200 mM NaCl, 5 mM DTT, and 1% NP-40 on ice for 20 min with vortexing every 5 min, followed by 14 K RPM centrifugation at 4 °C for 10 min. 20 μg total protein was incubated with PNGase F (New England Biolabs, Ipswich, MA) for 24 hrs. at 37 °C according to manufacturer’s instructions. After reaction was complete, samples were analyzed by PAGE NIR analysis using a LI-COR Odyssey CLx (LI-COR, Lincoln, NE)^22,28,50^

### Lentil lectin affinity purification

Cells were lysed in 20 mM Tris-HCl (pH 8), 200 mM NaCl, 5 mM DTT, and 1% NP-40 on ice for 20 min. with vortexing every 5 min., followed by 14 K RPM centrifugation at 4 °C for 10 min. The soluble fraction was incubated with Lentil Lectin Sepharose 4B beads (GE Healthcare, Chicago, IL) and 1 μL of 25 μL BG-782 for 1 hr at room temperature. Beads were pelleted and washed 3X in excess lysis buffer. Bound protein was eluted with lysis buffer supplemented with 200 mM methyl-α-D-mannopyranoside at 37 °C shaken at 300 RPM for 10 min. Elutant was collected and subjected to SDS-PAGE electrophoresis, followed by PAGE NIR.

### Tunicamycin treatment

HEK293 cells were transfected with WT SNAP-α_1D_. 24 hr after transfection, cells were washed 3X with PBS, and incubated with media containing 5 ug/mL of tunicamycin or 95% EtOH vehicle for 16 hr. Following incubation, cells were washed 3X with ice cold PBS, lysed, subjected to SDS PAGE, and analyzed by PAGE NIR as described above.

### Bortezomib treatment

HEK293 cell were transfected with either S-WT-C or S-NQQ-C. 24 hr after transfections, cells were treated with 1 μM of bortezomib or DMSO vehicle for 24 hr. Following treatment, cells were lysed and lysate analyzed via PAGE NIR.

### Protease inhibitor treatment

HEK293 cell were transfected with either S-WT-C or S-NQQ-C. 24 hr after transfections, cells were treated with of Pierce Protease Inhibitor cocktail (Thermo Scientific, Rockford, IL) using a 1:200 dilution or vehicle for 24 hr. Cells were then lysed and lysates were analyzed using PAGE NIR analysis.

### SNAP MS/MS

HEK293 cells were transiently transfected with either WT or NQQ SNAP-α_1D_ for 48 hrs. Cells were collected and lysed as above, with the addition of end-over-end rocking for 2 hrs. at 4 °C prior to centrifugation to remove insoluble fraction. Each condition was divided into 4 1.5 mL tubes for 16 hr. incubation with 20 μL of packed SNAP-Capture pull-down resin (New England Biolabs, Ipswich, MA) at 4 °C with end-over-end rocking. Beads were washed 3X with lysis buffer, transferred to new 1.5 mL tubes and washed 3X with 20 mM Tris-HCl pH 8.0 and 2 mM CaCl_2_. After the final wash the saturated beads were incubated with 20 mM Tris-HCl pH 8.0 supplemented with 5 mM DTT for 30 min at 60 °C with agitation, followed by incubation with 15 mM iodoacetamide for 10 min at RT. Denatured protein was then incubated with 1.5 μg of Trypsin (Sigma, St. Louis, MO) and 1.5 μg of Glu-C endoprotease (Thermo Fisher Scientific, Waltham, MA) for 16 hr at 37 °C with vigorous agitation. Peptides were collected and acidified using formic acid (FA) to a final concentration of 1% FA and desalted using StageTips^80^. Peptides were eluted from StageTips using elution buffer (40% acetonitrile, 1% FA), dried down and re-suspended in 8% acetonitrile, 1% FA. Samples were then loaded on a self-pulled 360 µm OD x 100 µm ID 15 cm column with a 7 µm tip packed with 3 µm Reprosil C18 resin (Dr. Maisch, Germany). Peptides were analyzed by nanoLC-MS in a 90 minutes linear gradient from 6% to 38% buffer B (buffer A: 0.1% acetic acid; buffer B: 0.1% acetic acid, 80% acetonitrile) on an EASY nLC 1200 (Thermo Scientific, Rockford, IL) and Orbitrap Fusion Lumos Tribrid Mass Spectrometer (FTMS; Thermo Scientific, Rockford, IL). Orbitrap FTMS spectra (R = 60 000 at 200 m/z; m/z 350–1600; 7e5 target; max 20 ms ion injection time) and Top Speed data-dependent acquisition with 3 second cycle time; HCD MS/MS spectra (R = 30 000 at 200 m/z; 31% CE; 5e4 target; max 100 ms injection time) were collected with an intensity filter set at 2.5e4 and dynamic exclusion for 45 second. Mass spectra were searched against the UniProt human reference proteome downloaded on February 20th, 2020 with the addition of SNAP-tag-ADRA1D sequence using MaxQuant v1.6.10.43. Detailed MaxQuant settings: samples were set to fraction 1 and 5 for NQQ mutant and WT, respectively, to allow within-group “match between run”; Trypsin/P and Glu-C were selected in digestion setting. Other settings were kept as default.

### Label free dynamic mass redistribution (DMR) assay

DMR assays were performed in 384-well Corning Epic microsensor plates (Corning, Corning, NY) using previously described protocols^22,28,50,81^. Data were analyzed using GraphPad Prism (La Jolla, CA).

### Cell surface assay

Cell surface assay was performed as described previously^22,28,50^.

### Sucrose density centrifugation

Cells (~6.7 M cells/mL) were suspended in detergent-free lysis buffer (1 mM Tris-HCl pH 7.4, 140 mm NaCl, 10% sucrose) on ice for 20 min with vortexing every 5 min. 19 μL of lysate (~125,000 cells) was labeled with BG-782 at 37 °C. Reacted lysate was gently layered on top of a discontinuous sucrose gradient. Gradient consisted of equal volumes of 65%, 62.5%, 60%, 57.5%, 55%, 52.5%, 50%, and 15% sucrose dissolved in 1 mM Tris-HCl pH 7.4 and 140 mM NaCl. Samples were centrifuged at 134,633 × *g* at 4 °C for 65 min using a TH-660 rotor (Thermo Fisher Scientific, Waltham, MA). 400 μL fractions were collected and subjected to PAGE NIR analysis. Fluorescence of each fraction was quantified (NIR: λ = 800 nm) using Image Studio software (LI-COR, Lincoln, NE) and analyzed using area under the curve (AUC) analysis with a cut-off of 10% and minimum change in height of 5% from minimum to maximum in GraphPad Prism (La Jolla, CA).

### Confocal microscopy

48 hours after transfection, cells were fixed with 4% paraformaldehyde/PBS solution for 10 min. at room temperature, washed with PBS, and permeabilized in 0.1% TritonX-100/PBS for 1 min. Cells were incubated with 1 μM of SNAP Surface Alexa Fluor 488 (New England BioLabs #S9129S, Ipswich, MA) and 1:1000 ER Staining Kit-Red Fluorescence-Cytopainter (Abcam #139482, Cambridge, MA) at 37 °C for 30 min. protected from light. Hoechst 33342 was used for nuclear staining. Cover slips were mounted using ProLong Glass antifade reagent (Thermo Fisher #P36982). Confocal fluorescence microscopy was performed using Leica SP8X laser scanning confocal microscope equipped with a 40x oil immersion objective (Leica Camera, Wetzlar, Germany). The detection pinhole was set to 1 Airy unit, light collection configuration was optimized according to the combination of chosen fluorochromes (Alexa Fluor 488, Texas Red, and Hoechst), and sequential channel acquisition was performed to minimize the risk of bleed-through. The intensity gain was adjusted for each channel before capture in order to avoid saturated pixels. 8 bit, 1024 × 1024 pixel images were collected as Z-stack acquisition. All microscopy was performed in collaboration with the W.M. Keck Microscopy center on the University of Washington School of Medicine campus.

### Colocalization analysis

The Alexa Fluor 488 (SNAP) and Texas Red (ER) channels were analyzed for colocalization using Coloc2 plugin for Fiji^82^. Pearson’s coefficients for a cell were averaged over each slice in a Z-stack. Data were analyzed using GraphPad Prism (La Jolla, CA).

## Supplementary information

Supplementary information.

Supplementary Data 1

Supplementary Data 2
