# Supplementary material for: N-glycosylation of α1D-adrenergic receptor N-terminal domain is required for correct trafficking, function, and biogenesis
Source: Sci Rep. 2020 Apr 29;10:7209. doi: 10.1038/s41598-020-64102-4 (PMC7190626; doi:10.1038/s41598-020-64102-4)
Supplement: Supplementary file 1 — Supplementary information. [file 41598_2020_64102_MOESM1_ESM.docx]

***N­-*glycosylation of α_1D_-adrenergic receptor N-terminal domain is required for correct trafficking, function, and biogenesis**

Eric M. Janezic^1§^, Sophia My-Linh Lauer^1^, Robert George Williams^1^, Michael Chungyoun^1^, Kyung-Soon Lee^1^, Edelmar Navaluna^1^, Ho-Tak Lau^1^, Shao-En Ong^1^, Chris Hague^1*^

^1^Department of Pharmacology, School of Medicine, University of Washington, 1959 NE Pacific Street, Seattle, WA 98185, USA

^§^ORCID ID = 0000-0002-3177-889X

*To whom correspondence should be addressed: Chris Hague, Dept. of Pharmacology, University of Washington School of Medicine, 1959 NE Pacific Ave. Box 357280, Seattle, WA 98195; Email: chague@uw.edu; Tel. (206) 221-4612


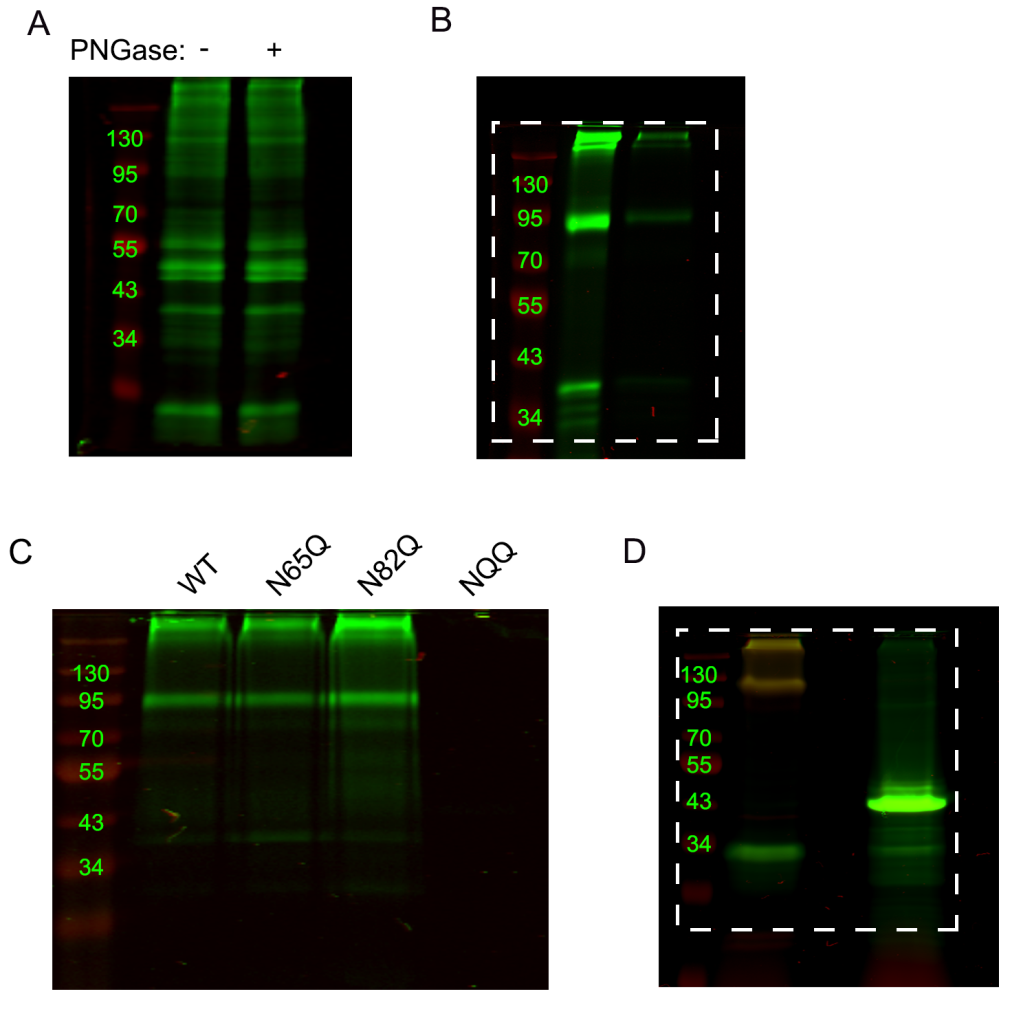


**Supplementary Figure S1. Supplementary data for Figure 1.** (A) HEK293 cells were transiently transfected with WT SNAP-α_1D_ and lysates were subjected to vehicle (-) or PNGase F (+) deglycosylation assay followed by PAGE NIR analysis. (B) Uncropped PAGE NIR gel from figure 1A. White rectangle delineates cropping. (C) WT, N65Q, and N82Q SNAP-α_1D_, but not NQQ, interact with lentil lectin beads. (D) Uncropped gel from figure 1E. White rectangle signifies cropped area displayed in figure 1E.


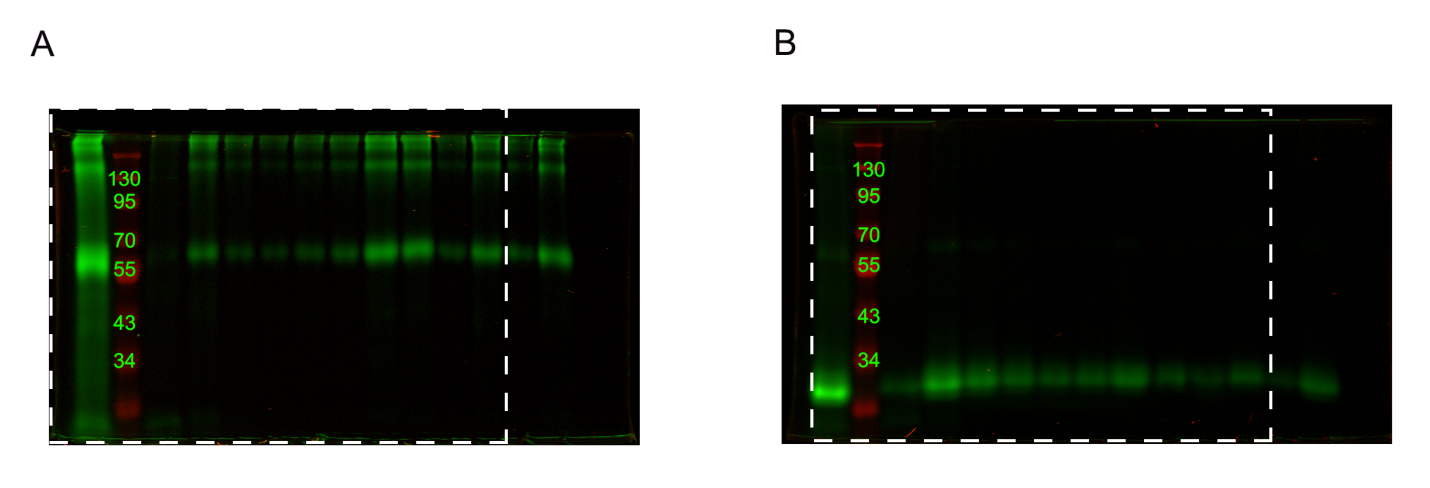


**Supplementary Figure S2. Uncropped SDS PAGE gels corresponding to figure 4.**  (A) Uncropped image from figure 4A. (B) Uncropped SDS PAGE gel for figure 4B. White rectangle denoted where images were cropped.


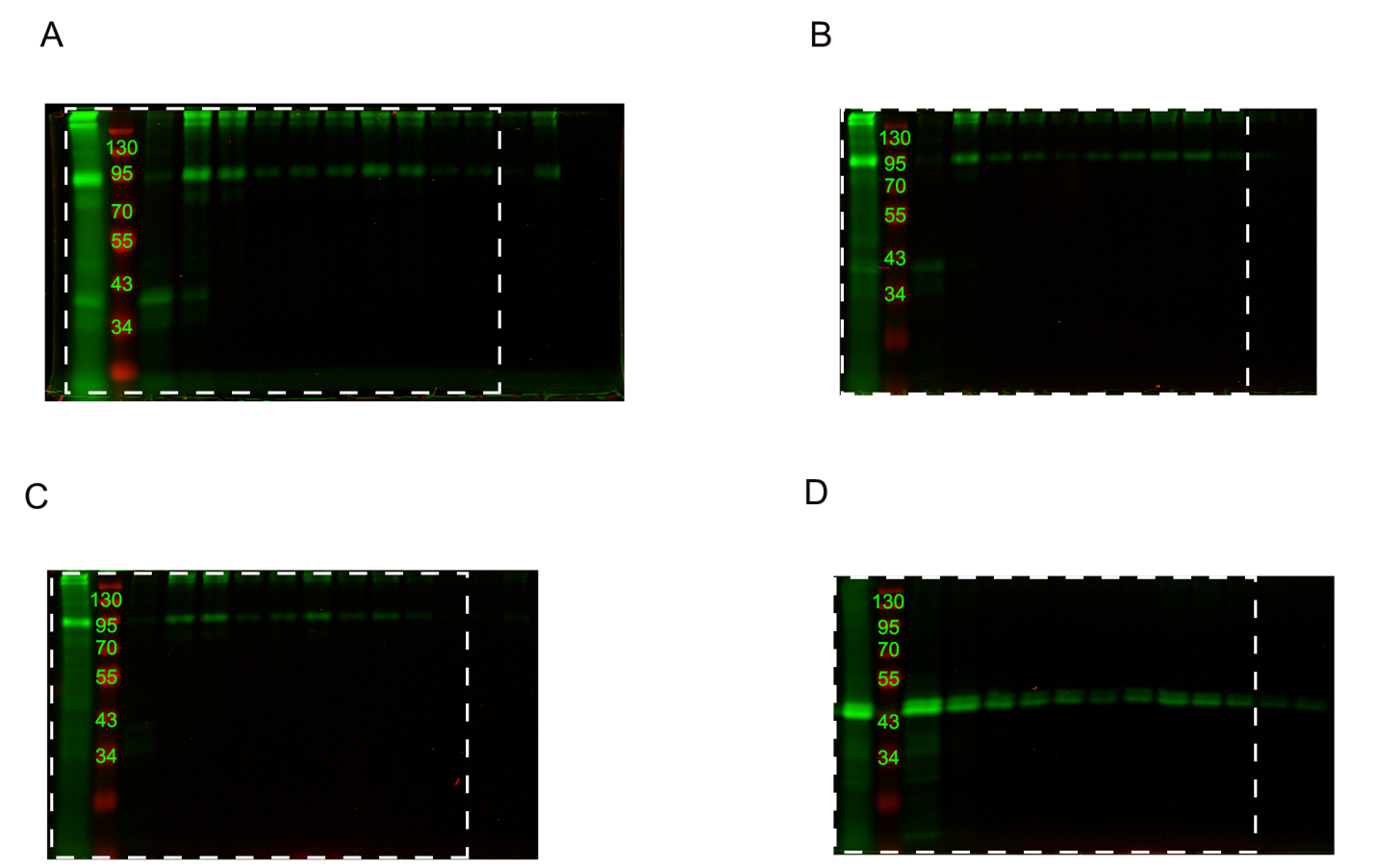


**Supplementary Figure S3. Uncropped SDS PAGE gels for figure 5.** (A) Uncropped image corresponding to figure 5A. (B) Full gel for figure 5B. (C) Uncropped SDS PAGE gel for figure 5C. (D) Uncropped image for figure 5D. White rectangle denotes cropped lines used in main text figures.


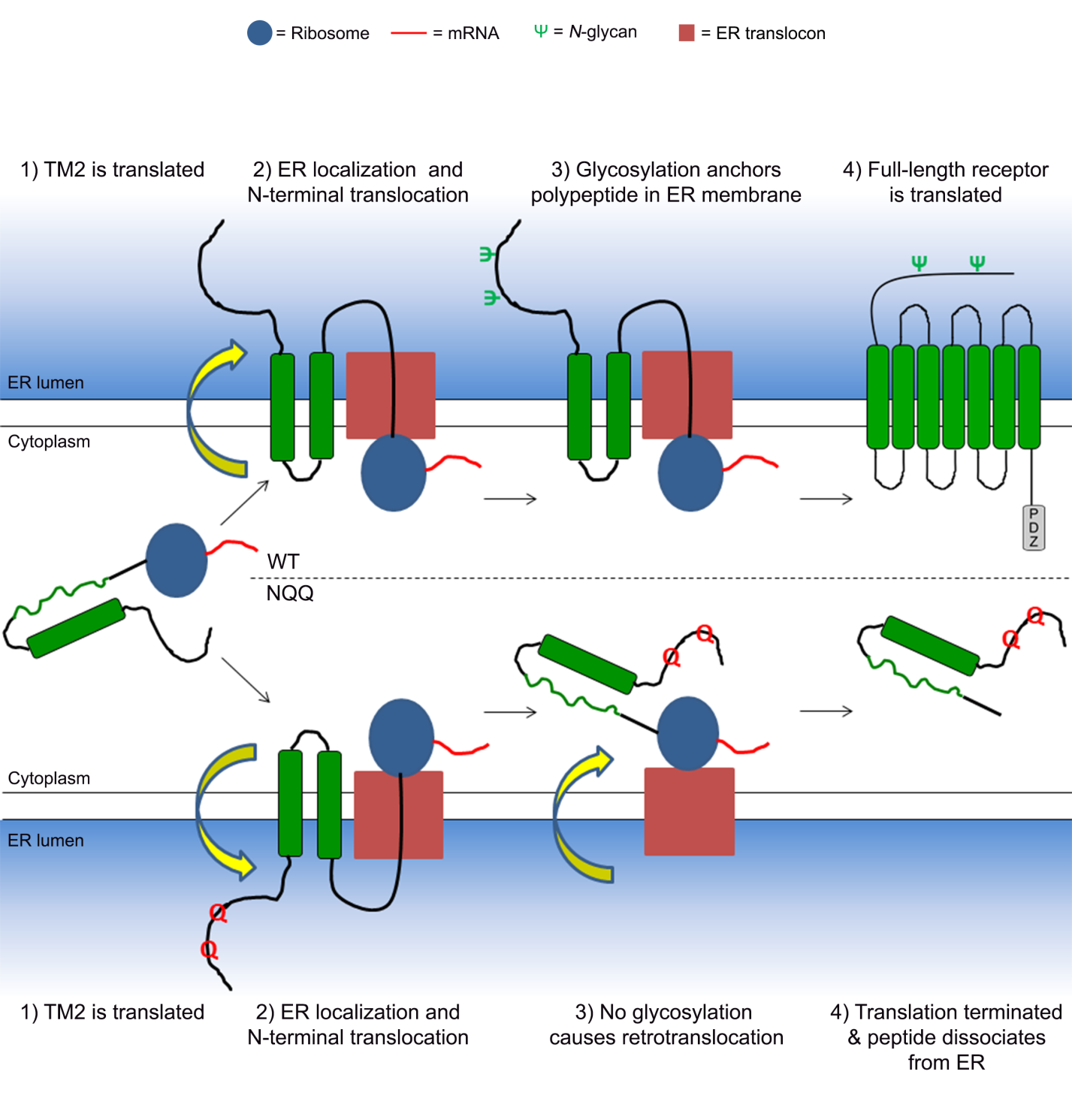


**Supplementary Figure S4. Model of proposed role for N-terminal glycosylation on α_1D_-AR biosynthesis.** *Top, WT:* ­­(1) Nascent α_1D_-AR peptide is translated in the cytoplasm through TM2. (2) Translation is halted as polypeptide, mRNA, and ribosomal complex translocate to the ER, where the N-terminal translocates into the ER lumen. (3) *N*-glycans anchor the immature receptor into the proper topology and ER membrane. (4) Translation continues. *Bottom, NQQ*: (1) Nascent α_1D_-AR peptide is translated in cytoplasm through TM2. (2) The translation is halted and the ribosome, mRNA, and peptide complex translocates to ER where N-terminal enters ER lumen. (3) Lack of glycosylation causes N-terminal to retrotranslocate. (4) Translation of α_1D_-AR is terminated and peptide dissociates from ER to likely be degraded.
